# Mortality trends in patients with coexisting pulmonary embolism and obesity: a major public health concern

**DOI:** 10.1016/j.rpth.2025.103338

**Published:** 2025-12-29

**Authors:** Vânia M. Morelli

**Affiliations:** 1 Thrombosis Research Group, Department of Clinical Medicine, UiT – The Arctic University of Norway, Tromsø, Norway; 2 Thrombosis Research Center, Division of Internal Medicine, University Hospital of North Norway, Tromsø, Norway; 3 Discipline of Hematology, Hemotherapy and Cell Therapy, Department of Internal Medicine, University of São Paulo Medical School, São Paulo, Brazil

Venous thromboembolism (VTE), encompassing deep vein thrombosis and pulmonary embolism (PE), is a prevalent disease affecting approximately 1 in 12 individuals during their lifetime [1]. VTE is associated with serious short- and long-term complications, including recurrence, postthrombotic syndrome, post-PE syndrome, and death [2]. Time-trend studies show that the incidence of VTE has risen over the past decades, essentially driven by an increasing incidence of PE [3,4]. Several factors may contribute to the rising incidence of PE, including increased awareness, improved diagnostic strategies that lead to better rates of disease detection, and the increasing prevalence of major PE risk factors, among which obesity is one of the most relevant from a contemporary public health perspective. The prevalence of obesity, defined as a body mass index ≥ 30 kg/m^2^, has more than doubled since 1990 worldwide and has reached epidemic proportions according to the World Health Organization [5]. It is worth noting that a genetically predicted elevated body mass index is associated with an increased risk of PE in Mendelian randomization studies [6], likely supporting a causal relationship between obesity and PE. According to a recent Norwegian population-based cohort study [7], the estimated population-attributable fraction of incident PE events due to overweight and obesity was nearly 28% (Figure). Given the increasing prevalence of obesity worldwide [5], it is reasonable to assume that the incidence of PE will continue to rise in the coming years.

**Figure:**
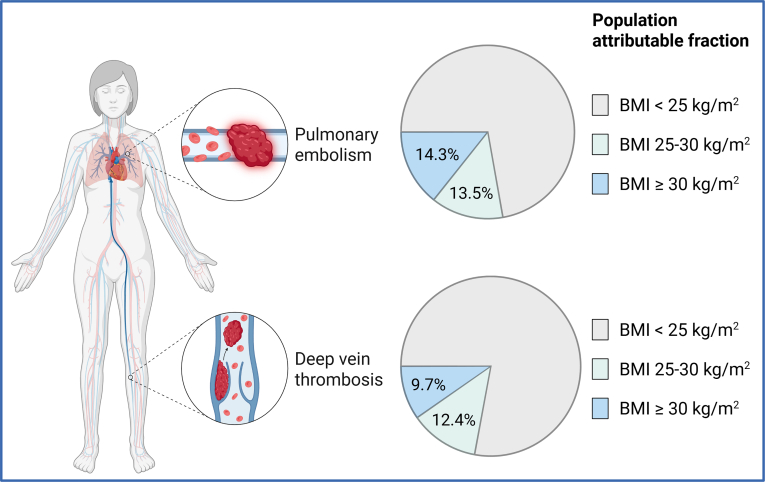
Population-attributable fraction adjusted for age and sex for pulmonary embolism and deep vein thrombosis, according to categories of body mass index (BMI) [7]. BMI 25 to 30 kg/m^2^: overweight; BMI ≥ 30 kg/m^2^: obese. (Created with BioRender.com).

Although obesity is a well-established risk factor for PE, the impact of obesity on clinical outcomes in patients with PE, particularly mortality, remains poorly understood. Some studies show lower mortality rates in obese patients compared with nonobese patients [[8], [9], [10]], findings that are in line with the “obesity paradox” [11]. Research focused on the interplay between obesity and clinical outcomes in patients with PE is crucial for identifying individuals at high risk of a poor prognosis, so that targeted interventions may be applied to mitigate mortality.

In this issue, Goyal et al. [12] shed more light on the interplay between obesity and PE-related mortality by examining mortality trends among people with coexisting PE and obesity in the United States over the past 2 decades and the influence of demographic factors on these trends. To this end, the authors used a nationwide database (the Centers for Disease Control and Prevention Wide-ranging Online Data for Epidemiologic Research) to assess the mortality rates among individuals aged ≥ 25 years with both PE and obesity from 1999 to 2020. During the study period, the age-adjusted mortality rate (AAMR) increased from 5.1 (95% CI, 4.7-5.4) per 1,000,000 individuals in 1999 to 13.9 (95% CI, 13.4-14.4) in 2020, with the rise most pronounced from 2018 to 2020.

What was the impact of demographic factors on mortality rates in people with coexisting PE and obesity? During the study period, the AAMRs per 1,000,000 people were consistently higher in women (8.8; 95% CI, 8.6-8.9) than in men (6.5; 95% CI, 6.4-6.6), and among the age groups, middle-aged adults (45-64 years) had higher AAMRs (10.0; 95% CI, 9.8-10.1) than older (≥ 65 years) and younger (25-44 years) adults. Among the ethnoracial groups, non-Hispanic Black people had the highest AAMRs per 1,000,000 people (16.8; 95% CI, 16.5-17.2). Moreover, the AAMRs were higher among residents of nonmetropolitan areas (8.9; 95% CI, 8.7-9.1) than among those living in metropolitan areas (7.5; 95% CI, 7.4-7.6).

The authors also investigated AAMRs in the United States from 1999 to 2020 related to PE or obesity alone [12]. Notably, throughout the study period, the increase in AAMRs was significantly greater for combined PE and obesity than for PE alone. As discussed by the authors, the rise in mortality from combined PE and obesity closely paralleled the upward trajectory of obesity-related mortality, suggesting that the obesity burden was a major driver of this trend.

Obesity is a complex chronic disease, and its prevalence has increased not only in the United States but also worldwide over the past decades, resulting in a global public health crisis, with approximately 1 billion people living with obesity in 2022, according to the World Health Organization [5]. The substantial rise in mortality rates from concurrent PE and obesity during the last 2 decades reported by Goyal et al. [12] is consistent with the marked increase in the prevalence of obesity [13] and obesity-related mortality in the United States [14]. Further, the pronounced mortality rates observed in women, non-Hispanic Black individuals, and residents of nonmetropolitan areas potentially reflect socioeconomic and healthcare access factors affecting this population.

What are the future perspectives? Goyal et al. [12] revealed a concerning upward trend in mortality rates among people with coexisting PE and obesity between 1999 and 2020, particularly in some demographic groups. The results of this US nationwide study contribute to increasing awareness of the need for targeted interventions in this high-risk population and form the basis for future studies aimed at investigating the explanatory factors for the rising mortality rates in obese people with PE.
